# Medical certification of cause of death for COVID-19

**DOI:** 10.2471/BLT.20.257600

**Published:** 2020-05-01

**Authors:** Chalapati Rao

**Affiliations:** a Research School of Population Health, Australian National University, 62, Mills Road, Acton, Australian Capital Territory 2601, Australia.

Reliable measurement of cause-specific mortality, analyses of morbid conditions and assessment of available health resources and services are among the first principles of epidemiology and evidence-based population health policy. With the continuing increase in mortality from the corona virus disease 19 (COVID-19), mortality analysis could be valuable in addressing the current pandemic.

Accurate information is essential to understand the epidemic profile and natural history of COVID-19. Epidemic profiles can be constructed from characteristics of seropositive cases, evaluation of exposures and contact tracing, and clinical observations on patients, but such evidence is usually difficult to acquire at the population level. To guide data recording, compilation and analysis, the World Health Organization (WHO) has published protocols for countries to notify COVID-19 individual case reports as well as aggregated data of newly confirmed cases and deaths.^1^ These data are compiled into daily WHO situation reports, which describe cumulative and new cases and deaths by country, also graphed as an epidemic curve.^2^

National governments are focusing on data collection of disease incidence and spread to inform epidemic control strategies. However, a need for greater focus on disease outcomes, particularly for deaths, also exists, given the rising mortality burden. COVID-19 mortality is being closely monitored at population level, but publicly available information lacks details on age and sex distributions or on the extent and nature of the co-morbidities. However, even the first basic mortality analysis from Italy during the first month of observations provides critical evidence on the higher proportions of males older than 70 years and of presence of multiple co-morbidities in COVID-19 deaths, although these were not specified in detail.^3^

More importantly though, the Italian analysis discusses the key issue of defining COVID-19 deaths. For the moment, considering deaths with test positivity as COVID-19 deaths could be appropriate, but these deaths should be reported with all other existing medical conditions, to enable more detailed analyses of causality or association. In practice, WHO’s *International statistical classification of diseases and related health problems* (ICD) includes a form for medical certification of cause of death.^4^ The form requires certifying physicians to record a pathophysiological sequence of clinical conditions leading to death, their durations and other contributory causes. For COVID-19 cases, the complete sequence, along with all other conditions co-existent with COVID-19 infection at death, would be required for detailed descriptive and analytical epidemiology. Analysis of clinical sequences from the medical certification of cause of death forms with chronology can be useful to guide priorities and resource allocation for critical care management, as well as enhance our understanding of epidemiological patterns and causal pathways to mortality from COVID-19. In addition, spatial and risk factor analysis of the forms can guide policy decisions on public health measures, such as personal protection, community quarantine and other suppression or mitigation strategies to control the spread of COVID-19.

All current epicentres of the pandemic routinely use WHO’s form for medical certification of cause of death. Certain additional variables could enhance the analytical value of COVID-19 mortality data, including the likely mode of infection, location (in health facilities or in the community), and use of specialist health services, among others. Therefore, a multipronged approach could be adopted for data acquisition, potentially through the network of WHO regional and country offices.^5^ First, all retrospective data from previously completed forms should be compiled in each country, that is, data for all COVID-19 deaths since the start of the pandemic. Second, epidemiologists should review case records and complete a modified form for at least a sample of previous deaths, to verify and supplement the original data. Third, the modified form should be implemented prospectively, with guidance to health-care personnel for ensuring data completeness and quality.^6^

These measures should be supported with an analysis protocol for uniform minimum data reporting across countries. The protocol should include an unambiguous definition of a COVID-19 death and instructions for listing COVID-19 infection within the causal sequence or as a contributory cause. Statistical analysis could include age-sex-location tabulations of deaths, proportionate distributions of underlying causes and terminal complications of COVID-19, and frequencies of common associations of COVID-19 with contributory causes. If reported, case fatality and other mortality rates should be age-standardized, to enable appropriate comparison.^3^ Epidemiologists would further analyse the mortality data from their settings and derive meaningful inferences. For better epidemic surveillance, strengthening medical certification of cause of death is needed across most countries in the world.^7^ Mortality analysis is a cornerstone of epidemiology, and being relatively straightforward, mortality analysis should be fully exploited for guidance in addressing the currently accelerating COVID-19 pandemic.
